# Trimethyl 1-(2-methyl-1-phenylsulfonyl-1*H*-indol-3-yl)propane-1,2,3-tricarbox­ylate

**DOI:** 10.1107/S1600536808043286

**Published:** 2009-01-08

**Authors:** T. Kavitha, M. Thenmozhi, Radhakrishnan Sureshbabu, A. K. Mohanakrishnan, M. N. Ponnuswamy

**Affiliations:** a Centre of Advanced Study in Crystallography and Biophysics, University of Madras, Guindy Campus, Chennai 600025, India; b Department of Organic Chemistry, University of Madras, Guindy Campus, Chennai 600025, India.

## Abstract

In the title compound, C_24_H_25_NO_8_S, the indole unit is planar and makes a dihedral angle of 79.73 (11)° with the phenyl ring of the sulfonyl substituent. The mol­ecules in the unit cell are stabilized by C—H⋯O and C—H⋯π inter­molecular inter­actions in addition to van der Waals forces.

## Related literature

For the bological activity and applications of indole derivatives, see: Ho *et al.* (1986 ▶); Rajeswaran *et al.* (1999 ▶); Stevenson *et al.* (2000 ▶). For the Thorpe-Ingold effect, see: Bassindale (1984 ▶). For bond-length data, see: Allen *et al.* (1987 ▶).
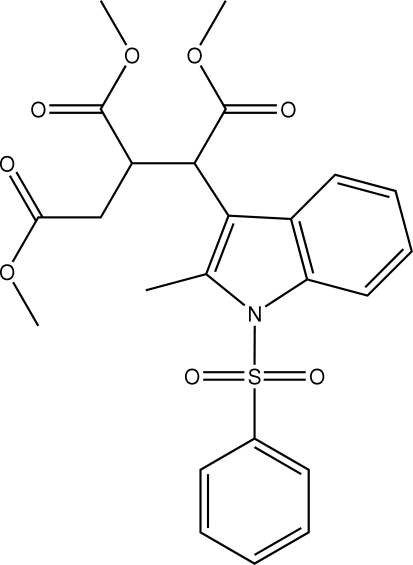


## Experimental

### 

#### Crystal data

C_24_H_25_NO_8_S
*M* *_r_* = 487.51
Monoclinic, 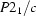

*a* = 11.0975 (3) Å
*b* = 9.8255 (3) Å
*c* = 22.0027 (6) Å
β = 98.605 (2)°
*V* = 2372.13 (12) Å^3^
*Z* = 4
Mo *K*α radiation
μ = 0.19 mm^−1^
*T* = 293 (2) K
0.40 × 0.30 × 0.15 mm

#### Data collection

Bruker Kappa APEXII diffractometer
Absorption correction: multi-scan (*SADABS*; Sheldrick, 2001 ▶) *T*_min_ = 0.835, *T*_max_ = 0.973
25507 measured reflections
5592 independent reflections
3885 reflections with *I* > 2σ(*I*)
*R*_int_ = 0.027

#### Refinement

*R*[*F* ^2^ > 2σ(*F* ^2^)] = 0.046
*wR*(*F* ^2^) = 0.135
*S* = 1.02
5592 reflections
308 parameters
1 restraint
H-atom parameters constrained
Δρ_max_ = 0.25 e Å^−3^
Δρ_min_ = −0.27 e Å^−3^

### 

Data collection: *APEX2* (Bruker, 2004 ▶); cell refinement: *SAINT* (Bruker, 2004 ▶); data reduction: *SAINT*; program(s) used to solve structure: *SIR92* (Altomare *et al.*, 1993 ▶); program(s) used to refine structure: *SHELXL97* (Sheldrick, 2008 ▶); molecular graphics: *ORTEP* (Farrugia, 1997 ▶); software used to prepare material for publication: *SHELXL97* and *PLATON* (Spek, 2003 ▶).

## Supplementary Material

Crystal structure: contains datablocks I, rsb357. DOI: 10.1107/S1600536808043286/bt2840sup1.cif

Structure factors: contains datablocks I. DOI: 10.1107/S1600536808043286/bt2840Isup2.hkl

Additional supplementary materials:  crystallographic information; 3D view; checkCIF report

## Figures and Tables

**Table 1:** Hydrogen-bond geometry (Å, °)

*D*—H⋯*A*	*D*—H	H⋯*A*	*D*⋯*A*	*D*—H⋯*A*
C23—H23*A*⋯O3^i^	0.97	2.57	3.476 (3)	156
C13—H13⋯*Cg*1^ii^	0.93	2.73	3.633 (3)	163

Symmetry codes: (i) 
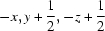
; (ii) 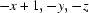. *Cg*1 is the centroid of the N1/C2–C5 ring.

**Fig. 1.**
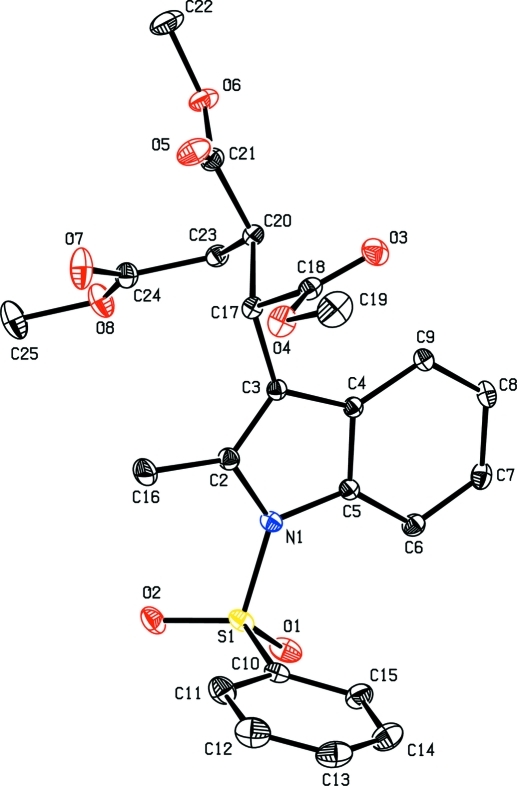
ORTEP plot of the molecule with displacement ellipsoids drawn at 20% probability level. H atoms have been omitted for clarity.

**Fig. 2.**
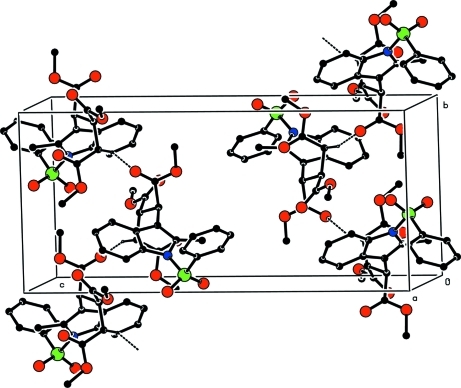
The molecular packing of the compound viewed down the b axis is shown. Dashed lines indicate hydrogen bonds. H atoms not involved in hydrogen bonding have been omitted.

## supplementary crystallographic information

### Comment

Indole derivatives are used as bioactive drugs (Stevenson *et al.*, 2000) and they exibit anti-allergic, central nervous system depressant and muscle relaxant properties (Ho *et al.*, 1986). Indoles also have been proved to display high aldose reductase inhibitory activity (Rajeswaran *et al.*, 1999).

The bond lengths N1- C2 [1.421 (2) Å] and N1- C5 [1.408 (2) Å] are longer than the mean value of 1.355 (1) Å reported for the indole moiety (Allen *et al.*, 1987). The S atom shows a distorted tetrahedral geometry, with O1—S1—O2 [119.99 (11)°] and N1—S1—C10 [105.05 (9)°] angles deviating from ideal tetrahedral values, are attributed to the Thorpe-Ingold effect (Bassindale, 1984). The indole ring is planar and the sulfonyl bound phenyl ring is almost perpendicular to the indole ring system, with a dihedral angle of 79.73 (11)°. The sum of bond angles around N1 (359.5°) indicates that N1 is in *sp*^2^ hybridization. The ester groups attached to the indole ring system assume extended conformation [C23- C24- O8- C25 = -178.8 (2)°; C20- C21- O6- C22 = -179.2 (2)° and C17- C18- O4- C19 = 177.7 (2)°].

The molecules in the crystal structure extend along the *b* axis *via* intermolecular C—H···O hydrogen bonds involving atoms C23 and O3 (-*x*,*y* + 1/2,- *z* + 1/2). Dimerization of the molecules occurs through C—H···π interactions [C13—H13 =0.93, H13···*Cg*1 = 2.7340, C13···*Cg*1 = 3.633 (3) Å and C13—H13···*Cg*1 = 162.73 °, where *Cg*1 is the centroid of the ring N1—C5].

### Experimental

To a stirred solution of dimethyl 2-(2-methyl-1-(phenylsulfonyl)-1*H*-indol-3-yl) maleate (0.4 g, 0.96 mmol) in dry DMF (1.5 ml), dimethyl acetamide dimethyl acetal (257 mg, 1.9 mmol) was added. Reaction mixture was heated to 110° C for 3hrs under nitrogen atmosphere. Then it was poured to 2% aqueous HCl (15 ml) solution and extracted with CHCl_3_. Organic layer was dried over Na_2_SO_4_ and evaporated. The crude compound was recrystallized from methanol.

### Refinement

H atoms were positioned geometrically (C—H = 0.93–0.98 Å) and allowed to ride on their parent atoms, with *U*_iso_(H) = 1.5*U*_eq_(C_methyl_) and 1.2*U*_eq_(C). The components of the anisotropic displacement parameters of C11 and C12 in the direction of the bond between them were restrained to be equal within an effective standard deviation of 0.001.

### Crystal data

**Table:**

C_24_H_25_NO_8_S	*F*(000) = 1024
*M**_r_* = 487.51	*D*_x_ = 1.365 Mg m^−^^3^
Monoclinic, *P*2_1_/*c*	Mo *K*α radiation, λ = 0.71073 Å
Hall symbol: -P 2ybc	Cell parameters from 5592 reflections
*a* = 11.0975 (3) Å	θ = 2.3–27.8°
*b* = 9.8255 (3) Å	µ = 0.19 mm^−^^1^
*c* = 22.0027 (6) Å	*T* = 293 K
β = 98.605 (2)°	Needle, colourless
*V* = 2372.13 (12) Å^3^	0.40 × 0.30 × 0.15 mm
*Z* = 4	

### Data collection

**Table:**

Bruker KAPPA APEXII diffractometer	5592 independent reflections
Radiation source: fine-focus sealed tube	3885 reflections with *I* > 2σ(*I*)
graphite	*R*_int_ = 0.027
ω and φ scan	θ_max_ = 27.8°, θ_min_ = 2.3°
Absorption correction: multi-scan (*SADABS*; Sheldrick, 2001)	*h* = −14→14
*T*_min_ = 0.835, *T*_max_ = 0.973	*k* = −12→12
25507 measured reflections	*l* = −28→28

### Refinement

**Table:**

Refinement on *F*^2^	Primary atom site location: structure-invariant direct methods
Least-squares matrix: full	Secondary atom site location: difference Fourier map
*R*[*F*^2^ > 2σ(*F*^2^)] = 0.046	Hydrogen site location: inferred from neighbouring sites
*wR*(*F*^2^) = 0.135	H-atom parameters constrained
*S* = 1.01	*w* = 1/[σ^2^(*F*_o_^2^) + (0.0566*P*)^2^ + 0.8871*P*] where *P* = (*F*_o_^2^ + 2*F*_c_^2^)/3
5592 reflections	(Δ/σ)_max_ < 0.001
308 parameters	Δρ_max_ = 0.25 e Å^−^^3^
1 restraint	Δρ_min_ = −0.27 e Å^−^^3^

### Special details

**Table:**

Geometry. All e.s.d.'s (except the e.s.d. in the dihedral angle between two l.s. planes) are estimated using the full covariance matrix. The cell e.s.d.'s are taken into account individually in the estimation of e.s.d.'s in distances, angles and torsion angles; correlations between e.s.d.'s in cell parameters are only used when they are defined by crystal symmetry. An approximate (isotropic) treatment of cell e.s.d.'s is used for estimating e.s.d.'s involving l.s. planes.
Refinement. Refinement of *F*^2^ against ALL reflections. The weighted *R*-factor *wR* and goodness of fit *S* are based on *F*^2^, conventional *R*-factors *R* are based on *F*, with *F* set to zero for negative *F*^2^. The threshold expression of *F*^2^ > σ(*F*^2^) is used only for calculating *R*-factors(gt) *etc*. and is not relevant to the choice of reflections for refinement. *R*-factors based on *F*^2^ are statistically about twice as large as those based on *F*, and *R*- factors based on ALL data will be even larger.

### Fractional atomic coordinates and isotropic or equivalent isotropic displacement parameters (Å^2^)

**Table:**

	*x*	*y*	*z*	*U*_iso_*/*U*_eq_	
C2	0.20679 (17)	0.1921 (2)	0.08315 (8)	0.0503 (4)	
C3	0.19449 (15)	0.11193 (18)	0.13178 (8)	0.0433 (4)	
C4	0.29851 (15)	0.13314 (17)	0.17830 (8)	0.0418 (4)	
C5	0.37387 (16)	0.22937 (18)	0.15615 (8)	0.0433 (4)	
C6	0.48157 (17)	0.2729 (2)	0.19067 (9)	0.0539 (5)	
H6	0.5319	0.3352	0.1750	0.065*	
C7	0.51138 (19)	0.2207 (2)	0.24880 (10)	0.0604 (5)	
H7	0.5826	0.2495	0.2731	0.072*	
C8	0.4380 (2)	0.1263 (2)	0.27211 (9)	0.0600 (5)	
H8	0.4602	0.0934	0.3118	0.072*	
C9	0.33297 (17)	0.0807 (2)	0.23731 (8)	0.0508 (4)	
H9	0.2851	0.0154	0.2529	0.061*	
C10	0.46121 (19)	0.2643 (2)	0.00851 (9)	0.0540 (5)	
C11	0.4134 (3)	0.2268 (3)	−0.04999 (10)	0.0781 (7)	
H11	0.3367	0.2565	−0.0680	0.094*	
C12	0.4830 (4)	0.1428 (4)	−0.08160 (13)	0.1017 (10)	
H12	0.4531	0.1172	−0.1217	0.122*	
C13	0.5936 (3)	0.0974 (3)	−0.05521 (16)	0.0975 (9)	
H13	0.6381	0.0395	−0.0769	0.117*	
C14	0.6397 (3)	0.1361 (3)	0.00258 (16)	0.0978 (9)	
H14	0.7161	0.1052	0.0204	0.117*	
C15	0.5745 (2)	0.2208 (3)	0.03505 (12)	0.0742 (6)	
H15	0.6067	0.2483	0.0746	0.089*	
C16	0.1242 (2)	0.2032 (3)	0.02344 (10)	0.0815 (7)	
H16A	0.0582	0.1397	0.0228	0.122*	
H16B	0.1688	0.1831	−0.0096	0.122*	
H16C	0.0921	0.2940	0.0187	0.122*	
C17	0.08720 (16)	0.02092 (19)	0.13676 (9)	0.0485 (4)	
H17	0.0281	0.0318	0.0993	0.058*	
C18	0.12894 (18)	−0.1261 (2)	0.14060 (10)	0.0561 (5)	
C19	0.1671 (3)	−0.3195 (3)	0.08387 (17)	0.1092 (11)	
H19A	0.1599	−0.3499	0.0420	0.164*	
H19B	0.1187	−0.3768	0.1060	0.164*	
H19C	0.2508	−0.3241	0.1026	0.164*	
C20	0.02316 (16)	0.0556 (2)	0.19188 (9)	0.0493 (4)	
H20	0.0750	0.0199	0.2284	0.059*	
C21	−0.09641 (18)	−0.0204 (2)	0.18689 (11)	0.0588 (5)	
C22	−0.2746 (2)	−0.0409 (3)	0.23308 (17)	0.1057 (11)	
H22A	−0.3099	−0.0019	0.2663	0.159*	
H22B	−0.2635	−0.1370	0.2397	0.159*	
H22C	−0.3278	−0.0257	0.1951	0.159*	
C23	0.00794 (17)	0.2075 (2)	0.20302 (9)	0.0510 (4)	
H23A	−0.0237	0.2193	0.2415	0.061*	
H23B	0.0876	0.2503	0.2076	0.061*	
C24	−0.0740 (2)	0.2789 (2)	0.15365 (10)	0.0601 (5)	
C25	−0.1514 (3)	0.4926 (3)	0.11907 (15)	0.1146 (12)	
H25A	−0.1432	0.5867	0.1308	0.172*	
H25B	−0.2346	0.4647	0.1181	0.172*	
H25C	−0.1283	0.4809	0.0791	0.172*	
N1	0.31622 (14)	0.26844 (17)	0.09733 (7)	0.0501 (4)	
O1	0.45912 (16)	0.45716 (15)	0.08815 (7)	0.0753 (5)	
O2	0.27957 (16)	0.42841 (17)	0.00952 (7)	0.0764 (5)	
O3	0.16646 (16)	−0.18474 (16)	0.18733 (8)	0.0770 (5)	
O4	0.12431 (16)	−0.17999 (17)	0.08525 (8)	0.0783 (5)	
O5	−0.12856 (16)	−0.1090 (2)	0.15146 (9)	0.0887 (6)	
O6	−0.15876 (13)	0.02166 (17)	0.23022 (8)	0.0749 (5)	
O7	−0.13414 (19)	0.2252 (2)	0.11119 (9)	0.1051 (7)	
O8	−0.07383 (19)	0.41121 (17)	0.16292 (8)	0.0862 (5)	
S1	0.37776 (5)	0.37135 (5)	0.04991 (2)	0.05603 (16)	

### Atomic displacement parameters (Å^2^)

**Table:**

	*U*^11^	*U*^22^	*U*^33^	*U*^12^	*U*^13^	*U*^23^
C2	0.0531 (10)	0.0538 (11)	0.0440 (9)	0.0028 (8)	0.0067 (8)	−0.0011 (8)
C3	0.0434 (9)	0.0440 (9)	0.0427 (9)	0.0047 (7)	0.0073 (7)	−0.0023 (7)
C4	0.0428 (9)	0.0397 (9)	0.0436 (9)	0.0063 (7)	0.0084 (7)	−0.0009 (7)
C5	0.0472 (9)	0.0412 (9)	0.0422 (9)	0.0056 (7)	0.0090 (7)	−0.0009 (7)
C6	0.0508 (10)	0.0504 (11)	0.0605 (11)	−0.0032 (8)	0.0086 (9)	−0.0026 (9)
C7	0.0528 (11)	0.0553 (12)	0.0676 (13)	0.0011 (9)	−0.0090 (9)	−0.0034 (10)
C8	0.0663 (12)	0.0561 (12)	0.0525 (11)	0.0088 (10)	−0.0078 (9)	0.0088 (9)
C9	0.0538 (10)	0.0478 (10)	0.0500 (10)	0.0038 (8)	0.0050 (8)	0.0081 (8)
C10	0.0688 (12)	0.0471 (10)	0.0494 (10)	−0.0065 (9)	0.0200 (9)	0.0035 (8)
C11	0.0970 (18)	0.0868 (18)	0.0511 (12)	0.0039 (14)	0.0131 (11)	−0.0042 (11)
C12	0.131 (3)	0.112 (2)	0.0685 (16)	−0.0052 (19)	0.0349 (17)	−0.0295 (16)
C13	0.108 (2)	0.086 (2)	0.113 (2)	−0.0005 (17)	0.060 (2)	−0.0206 (18)
C14	0.0734 (17)	0.112 (2)	0.115 (2)	0.0110 (16)	0.0366 (17)	−0.009 (2)
C15	0.0618 (13)	0.0907 (18)	0.0722 (14)	−0.0026 (12)	0.0165 (11)	−0.0093 (13)
C16	0.0815 (16)	0.107 (2)	0.0499 (12)	−0.0113 (15)	−0.0089 (11)	0.0143 (13)
C17	0.0444 (9)	0.0502 (10)	0.0501 (10)	0.0013 (8)	0.0050 (8)	−0.0035 (8)
C18	0.0487 (10)	0.0503 (11)	0.0705 (13)	−0.0022 (9)	0.0124 (9)	−0.0050 (10)
C19	0.117 (2)	0.0646 (17)	0.148 (3)	0.0101 (16)	0.027 (2)	−0.0415 (19)
C20	0.0422 (9)	0.0526 (11)	0.0536 (10)	0.0018 (8)	0.0085 (8)	0.0017 (8)
C21	0.0488 (10)	0.0526 (12)	0.0760 (14)	0.0018 (9)	0.0121 (10)	0.0042 (11)
C22	0.0609 (14)	0.092 (2)	0.175 (3)	−0.0002 (14)	0.0522 (18)	0.017 (2)
C23	0.0485 (10)	0.0534 (11)	0.0521 (10)	−0.0001 (8)	0.0107 (8)	−0.0055 (9)
C24	0.0615 (12)	0.0592 (13)	0.0586 (12)	0.0057 (10)	0.0053 (10)	−0.0031 (10)
C25	0.156 (3)	0.079 (2)	0.099 (2)	0.031 (2)	−0.010 (2)	0.0267 (17)
N1	0.0567 (9)	0.0531 (9)	0.0413 (8)	−0.0020 (7)	0.0102 (7)	0.0041 (7)
O1	0.1135 (13)	0.0496 (9)	0.0662 (9)	−0.0231 (8)	0.0248 (9)	−0.0078 (7)
O2	0.0989 (12)	0.0637 (10)	0.0682 (9)	0.0233 (9)	0.0176 (9)	0.0220 (8)
O3	0.0903 (12)	0.0548 (9)	0.0878 (11)	0.0109 (8)	0.0190 (9)	0.0118 (9)
O4	0.0842 (11)	0.0650 (10)	0.0850 (11)	0.0087 (8)	0.0105 (9)	−0.0285 (9)
O5	0.0715 (10)	0.0827 (12)	0.1152 (14)	−0.0247 (9)	0.0244 (10)	−0.0281 (11)
O6	0.0568 (8)	0.0733 (10)	0.1017 (12)	−0.0007 (7)	0.0357 (8)	−0.0018 (9)
O7	0.1096 (15)	0.0845 (13)	0.1019 (14)	0.0232 (11)	−0.0467 (12)	−0.0187 (11)
O8	0.1247 (15)	0.0551 (9)	0.0718 (10)	0.0118 (10)	−0.0082 (10)	0.0064 (8)
S1	0.0792 (4)	0.0419 (3)	0.0498 (3)	0.0007 (2)	0.0190 (2)	0.0046 (2)

### Geometric parameters (Å, °)

**Table:**

C2—C3	1.352 (3)	C17—C18	1.516 (3)
C2—N1	1.421 (2)	C17—C20	1.533 (3)
C2—C16	1.489 (3)	C17—H17	0.9800
C3—C4	1.439 (2)	C18—O3	1.198 (3)
C3—C17	1.506 (3)	C18—O4	1.322 (3)
C4—C9	1.396 (2)	C19—O4	1.452 (3)
C4—C5	1.397 (2)	C19—H19A	0.9600
C5—C6	1.384 (3)	C19—H19B	0.9600
C5—N1	1.408 (2)	C19—H19C	0.9600
C6—C7	1.372 (3)	C20—C21	1.512 (3)
C6—H6	0.9300	C20—C23	1.526 (3)
C7—C8	1.382 (3)	C20—H20	0.9800
C7—H7	0.9300	C21—O5	1.187 (3)
C8—C9	1.371 (3)	C21—O6	1.325 (3)
C8—H8	0.9300	C22—O6	1.434 (3)
C9—H9	0.9300	C22—H22A	0.9600
C10—C11	1.368 (3)	C22—H22B	0.9600
C10—C15	1.373 (3)	C22—H22C	0.9600
C10—S1	1.745 (2)	C23—C24	1.484 (3)
C11—C12	1.386 (4)	C23—H23A	0.9700
C11—H11	0.9300	C23—H23B	0.9700
C12—C13	1.353 (4)	C24—O7	1.188 (3)
C12—H12	0.9300	C24—O8	1.316 (3)
C13—C14	1.352 (4)	C25—O8	1.436 (3)
C13—H13	0.9300	C25—H25A	0.9600
C14—C15	1.371 (4)	C25—H25B	0.9600
C14—H14	0.9300	C25—H25C	0.9600
C15—H15	0.9300	N1—S1	1.6706 (16)
C16—H16A	0.9600	O1—S1	1.4165 (16)
C16—H16B	0.9600	O2—S1	1.4148 (16)
C16—H16C	0.9600		
			
C3—C2—N1	108.53 (16)	C20—C17—H17	108.1
C3—C2—C16	128.23 (19)	O3—C18—O4	124.0 (2)
N1—C2—C16	123.25 (18)	O3—C18—C17	124.8 (2)
C2—C3—C4	108.26 (16)	O4—C18—C17	111.16 (19)
C2—C3—C17	125.32 (16)	O4—C19—H19A	109.5
C4—C3—C17	126.38 (16)	O4—C19—H19B	109.5
C9—C4—C5	118.63 (16)	H19A—C19—H19B	109.5
C9—C4—C3	133.41 (17)	O4—C19—H19C	109.5
C5—C4—C3	107.95 (15)	H19A—C19—H19C	109.5
C6—C5—C4	122.01 (17)	H19B—C19—H19C	109.5
C6—C5—N1	131.02 (17)	C21—C20—C23	112.17 (16)
C4—C5—N1	106.95 (15)	C21—C20—C17	109.78 (16)
C7—C6—C5	117.66 (19)	C23—C20—C17	114.83 (16)
C7—C6—H6	121.2	C21—C20—H20	106.5
C5—C6—H6	121.2	C23—C20—H20	106.5
C6—C7—C8	121.56 (19)	C17—C20—H20	106.5
C6—C7—H7	119.2	O5—C21—O6	124.3 (2)
C8—C7—H7	119.2	O5—C21—C20	125.7 (2)
C9—C8—C7	120.73 (18)	O6—C21—C20	109.94 (19)
C9—C8—H8	119.6	O6—C22—H22A	109.5
C7—C8—H8	119.6	O6—C22—H22B	109.5
C8—C9—C4	119.37 (18)	H22A—C22—H22B	109.5
C8—C9—H9	120.3	O6—C22—H22C	109.5
C4—C9—H9	120.3	H22A—C22—H22C	109.5
C11—C10—C15	121.2 (2)	H22B—C22—H22C	109.5
C11—C10—S1	119.50 (18)	C24—C23—C20	114.51 (17)
C15—C10—S1	119.32 (17)	C24—C23—H23A	108.6
C10—C11—C12	117.8 (3)	C20—C23—H23A	108.6
C10—C11—H11	121.1	C24—C23—H23B	108.6
C12—C11—H11	121.1	C20—C23—H23B	108.6
C13—C12—C11	121.3 (3)	H23A—C23—H23B	107.6
C13—C12—H12	119.4	O7—C24—O8	123.3 (2)
C11—C12—H12	119.4	O7—C24—C23	125.2 (2)
C14—C13—C12	120.2 (3)	O8—C24—C23	111.51 (18)
C14—C13—H13	119.9	O8—C25—H25A	109.5
C12—C13—H13	119.9	O8—C25—H25B	109.5
C13—C14—C15	120.4 (3)	H25A—C25—H25B	109.5
C13—C14—H14	119.8	O8—C25—H25C	109.5
C15—C14—H14	119.8	H25A—C25—H25C	109.5
C14—C15—C10	119.2 (3)	H25B—C25—H25C	109.5
C14—C15—H15	120.4	C5—N1—C2	108.28 (15)
C10—C15—H15	120.4	C5—N1—S1	124.53 (13)
C2—C16—H16A	109.5	C2—N1—S1	126.71 (13)
C2—C16—H16B	109.5	C18—O4—C19	115.4 (2)
H16A—C16—H16B	109.5	C21—O6—C22	117.8 (2)
C2—C16—H16C	109.5	C24—O8—C25	117.3 (2)
H16A—C16—H16C	109.5	O2—S1—O1	119.99 (11)
H16B—C16—H16C	109.5	O2—S1—N1	106.49 (9)
C3—C17—C18	109.37 (15)	O1—S1—N1	105.85 (8)
C3—C17—C20	113.00 (15)	O2—S1—C10	109.26 (10)
C18—C17—C20	109.98 (16)	O1—S1—C10	109.13 (10)
C3—C17—H17	108.1	N1—S1—C10	105.05 (9)
C18—C17—H17	108.1		
			
N1—C2—C3—C4	−1.3 (2)	C3—C17—C20—C23	41.5 (2)
C16—C2—C3—C4	178.3 (2)	C18—C17—C20—C23	164.05 (16)
N1—C2—C3—C17	176.42 (16)	C23—C20—C21—O5	139.6 (2)
C16—C2—C3—C17	−4.0 (3)	C17—C20—C21—O5	10.7 (3)
C2—C3—C4—C9	178.73 (19)	C23—C20—C21—O6	−43.5 (2)
C17—C3—C4—C9	1.1 (3)	C17—C20—C21—O6	−172.46 (17)
C2—C3—C4—C5	0.2 (2)	C21—C20—C23—C24	−60.8 (2)
C17—C3—C4—C5	−177.43 (16)	C17—C20—C23—C24	65.5 (2)
C9—C4—C5—C6	0.6 (3)	C20—C23—C24—O7	7.2 (3)
C3—C4—C5—C6	179.38 (16)	C20—C23—C24—O8	−173.73 (18)
C9—C4—C5—N1	−177.85 (15)	C6—C5—N1—C2	−179.95 (19)
C3—C4—C5—N1	0.92 (19)	C4—C5—N1—C2	−1.68 (19)
C4—C5—C6—C7	−1.6 (3)	C6—C5—N1—S1	7.5 (3)
N1—C5—C6—C7	176.41 (19)	C4—C5—N1—S1	−174.21 (12)
C5—C6—C7—C8	1.0 (3)	C3—C2—N1—C5	1.8 (2)
C6—C7—C8—C9	0.6 (3)	C16—C2—N1—C5	−177.8 (2)
C7—C8—C9—C4	−1.7 (3)	C3—C2—N1—S1	174.17 (14)
C5—C4—C9—C8	1.1 (3)	C16—C2—N1—S1	−5.4 (3)
C3—C4—C9—C8	−177.31 (19)	O3—C18—O4—C19	0.8 (3)
C15—C10—C11—C12	0.2 (4)	C17—C18—O4—C19	177.7 (2)
S1—C10—C11—C12	179.3 (2)	O5—C21—O6—C22	−2.2 (4)
C10—C11—C12—C13	1.1 (5)	C20—C21—O6—C22	−179.2 (2)
C11—C12—C13—C14	−1.5 (5)	O7—C24—O8—C25	0.3 (4)
C12—C13—C14—C15	0.5 (5)	C23—C24—O8—C25	−178.8 (2)
C13—C14—C15—C10	0.8 (5)	C5—N1—S1—O2	−156.71 (15)
C11—C10—C15—C14	−1.2 (4)	C2—N1—S1—O2	32.14 (19)
S1—C10—C15—C14	179.7 (2)	C5—N1—S1—O1	−27.97 (18)
C2—C3—C17—C18	116.4 (2)	C2—N1—S1—O1	160.89 (16)
C4—C3—C17—C18	−66.4 (2)	C5—N1—S1—C10	87.45 (16)
C2—C3—C17—C20	−120.8 (2)	C2—N1—S1—C10	−83.70 (18)
C4—C3—C17—C20	56.5 (2)	C11—C10—S1—O2	−13.0 (2)
C3—C17—C18—O3	88.7 (2)	C15—C10—S1—O2	166.18 (18)
C20—C17—C18—O3	−36.0 (3)	C11—C10—S1—O1	−145.95 (18)
C3—C17—C18—O4	−88.3 (2)	C15—C10—S1—O1	33.2 (2)
C20—C17—C18—O4	147.10 (17)	C11—C10—S1—N1	100.93 (19)
C3—C17—C20—C21	169.00 (15)	C15—C10—S1—N1	−79.91 (19)
C18—C17—C20—C21	−68.48 (19)		

### Hydrogen-bond geometry (Å, °)

**Table:**

*D*—H···*A*	*D*—H	H···*A*	*D*···*A*	*D*—H···*A*
C23—H23A···O3^i^	0.97	2.57	3.476 (3)	156
C13—H13···Cg1^ii^	0.93	2.73	3.633 (3)	163

Symmetry codes: (i) −*x*, *y*+1/2, −*z*+1/2; (ii) −*x*+1, −*y*, −*z*.

## References

[bb1] Allen, F. H., Kennard, O., Watson, D. G., Brammer, L., Orpen, A. G. & Taylor, R. (1987). *J. Chem. Soc. Perkin Trans. 2*, pp. S1–19.

[bb2] Altomare, A., Cascarano, G., Giacovazzo, C. & Guagliardi, A. (1993). *J. Appl. Cryst.* **26**, 343–350.

[bb3] Bassindale, A. (1984). *The Third Dimension in Organic Chemistry*, ch. 1, p. 11. New York: John Wiley and Sons.

[bb4] Bruker (2004). *SAINT* and *APEX2* . Bruker AXS Inc., Madison, Wisconsin, USA.

[bb5] Farrugia, L. J. (1997). *J. Appl. Cryst.* **30**, 565.

[bb6] Ho, C. Y., Haegman, W. E. & Perisco, F. (1986). *J. Med. Chem.* **29**, 118–121.

[bb7] Rajeswaran, W. G., Labroo, R. B., Cohen, L. A. & King, M. M. (1999). *J. Org. Chem.* **64**, 1369–1371.

[bb8] Sheldrick, G. M. (2001). *SADABS* University of Göttingen, Germany.

[bb9] Sheldrick, G. M. (2008). *Acta Cryst.* A**64**, 112–122. 10.1107/S0108767307043930 18156677

[bb10] Spek, A. L. (2003). *J. Appl. Cryst.* **36**, 7–13.

[bb11] Stevenson, G. I., Smith, A. L., Lewis, S. G., Neduvelil, J. G., Patel, S., Marwood, R. & Castro, J. L. (2000). *Bioorg. Med. Chem. Lett.* **10**, 2697–2704. 10.1016/s0960-894x(00)00557-6 11133071

